# A transfer learning nomogram for predicting prostate cancer and benign conditions on MRI

**DOI:** 10.1186/s12880-023-01163-7

**Published:** 2023-11-30

**Authors:** Junhao Chen, Bao Feng, Maoqing Hu, Feidong Huang, Yehang Chen, Xilun Ma, Wansheng Long

**Affiliations:** 1 Department of Medical Imaging Center, The First Affiliated Hospital of Jinan University, 613 West Huangpu Street, Tianhe District, Guangzhou, Guangdong Province 510630 PR China; 2 Department of Radiology, Jiangmen Central Hospital, Jiangmen, Guangdong Province 529000 PR China; 3 Laboratory of Artificial Intelligence of Biomedicine, Guilin University of Aerospace Technology, Guilin, Guangxi Province 541004 PR China; 4 School of Electronic Engineering and Automation, Guilin University of Electronic Technology, Guilin, Guangxi Province 541004 PR China; 5 Department of Radiology, The First Affiliated Hospital of Shantou University Medical College, Shantou, Guangdong Province 515000 PR China; 6 Department of Radiology, Jiangmen Central Hospital, 23#, North Road, Pengjiang Zone, Jiangmen, Guangdong Province 529000 PR China

**Keywords:** Prostatic cancer, Deep learning, Transfer learning, Magnetic resonance imaging

## Abstract

**Background:**

Deep learning has been used to detect or characterize prostate cancer (PCa) on medical images. The present study was designed to develop an integrated transfer learning nomogram (TLN) for the prediction of PCa and benign conditions (BCs) on magnetic resonance imaging (MRI).

**Methods:**

In this retrospective study, a total of 709 patients with pathologically confirmed PCa and BCs from two institutions were included and divided into training (*n* = 309), internal validation (*n* = 200), and external validation (*n* = 200) cohorts. A transfer learning signature (TLS) that was pretrained with the whole slide images of PCa and fine-tuned on prebiopsy MRI images was constructed. A TLN that integrated the TLS, the Prostate Imaging–Reporting and Data System (PI-RADS) score, and the clinical factor was developed by multivariate logistic regression. The performance of the TLS, clinical model (CM), and TLN were evaluated in the validation cohorts using the receiver operating characteristic (ROC) curve, the Delong test, the integrated discrimination improvement (IDI), and decision curve analysis.

**Results:**

TLS, PI-RADS score, and age were selected for TLN construction. The TLN yielded areas under the curve of 0.9757 (95% CI, 0.9613–0.9902), 0.9255 (95% CI, 0.8873–0.9638), and 0.8766 (95% CI, 0.8267–0.9264) in the training, internal validation, and external validation cohorts, respectively, for the discrimination of PCa and BCs. The TLN outperformed the TLS and the CM in both the internal and external validation cohorts. The decision curve showed that the TLN added more net benefit than the CM.

**Conclusions:**

The proposed TLN has the potential to be used as a noninvasive tool for PCa and BCs differentiation.

**Supplementary Information:**

The online version contains supplementary material available at 10.1186/s12880-023-01163-7.

## Background

In men, prostate cancer (PCa) is the most frequently diagnosed cancer in 112 of 185 countries globally and is the leading cause of cancer death in 48 countries [1]. The burden of PCa continues to increase in some developing countries [2]. An accurate diagnosis is the first step towards better management for patients with suspicious prostate abnormalities.

In the traditional PCa diagnostic pathway, patients with elevated serum prostate-specific antigen (PSA) and abnormal digital rectal examination (DRE) often undergo transrectal ultrasound-guided biopsy for PCa detection. However, it may cause unnecessary biopsies or missed PCa detection [3, 4]. In recent years, magnetic resonance imaging (MRI) has begun to play an essential role in the detection and diagnosis of PCa [5, 6], and multiparametric magnetic resonance imaging (mp-MRI) before biopsy has been recommended by clinical guidelines [7, 8]. The Prostate Imaging Reporting and Data System (PI-RADS) [9], as a guideline designed to standardize the acquisition, interpretation and reporting of prostate MRI, has been widely used in clinical practice and has shown good performance in the detection of PCa or clinically significant prostate cancer (csPCa) [10, 11]. However, a wide range of benign conditions (BCs) and anatomic patterns show overlapping characteristics with PCa on MRI [12, 13]. Even when utilizing PI-RADS, the assessment accuracy of PCa still varies across radiologists and requires much expertise and experience [14, 15]. In previous studies, the sensitivity and specificity for PCa diagnosis ranged from 73 to 100% and 8–100%, respectively [10]. For patients with PCa or BCs, the management strategies and treatments are different. Therefore, a more accurate and objective approach for PCa diagnosis before biopsy is needed.

Machine learning (ML) and deep learning (DL) applications in radiology have the potential to diminish inter-reader variability, improve radiology workflow and increase radiologist productivity [16]. In previous studies, the sensitivity and specificity for PCa diagnosis using ML ranged from 0.62 to 0.99 and 0.51 to 0.98, respectively [17]. Some studies have shown that the performance of ML and DL models for detecting or characterizing PCa on MRI was comparable to or better than that of some radiologists using PI-RADS [18–20]. However, there are some limitations; for example, some models lack external validation, and in ML and DL models, insufficient training examples may cause overfitting. Transfer learning (TL), as one of the strategies to solve overfitting [21, 22], has been adopted by some researchers for PCa detection [23] or classification [19] on MRI and performed better than deep learning or ML models without TL. However, there might be limitations in terms of generalizability due to the relatively small dataset scale or the lack of external validation.

The present study was designed to develop a prediction model incorporating MRI-based TL features, clinical MRI interpretation, and conventional clinical predictors to differentiate PCa from BCs.

## Methods

### Study population

**Fig. 1:**
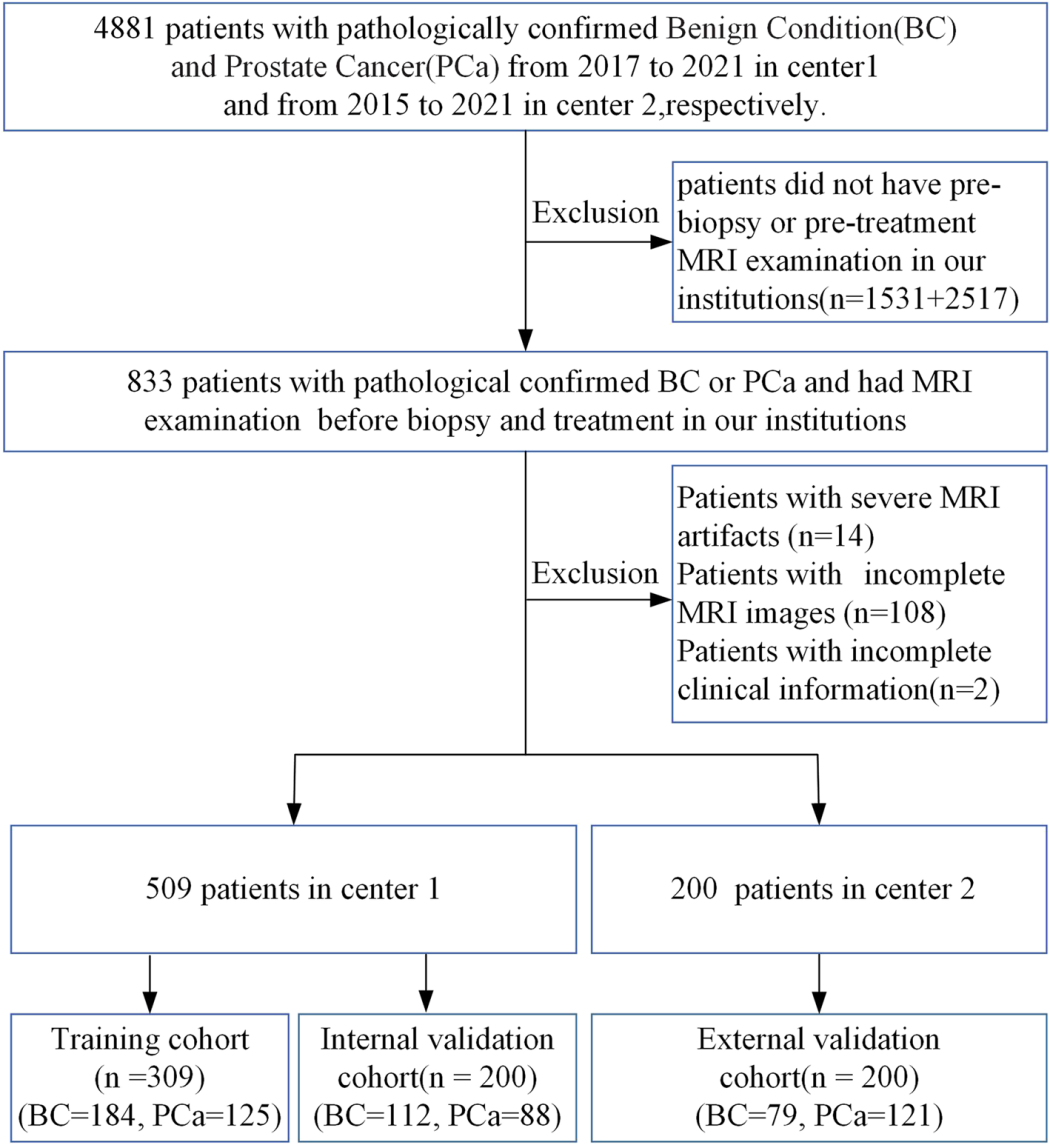
Flow chart of the study population

This retrospective study was approved by our institutional review board, and informed consent was waived. A total of 709 consecutive patients with pathologically proven PCa or BCs between 2015 and 2021 were enrolled from two institutions. The inclusion criteria were as follows: (a) pathologically confirmed primary prostate adenocarcinoma or benign conditions; (b) no radical prostatectomy, radiotherapy, hormonal therapy, or other therapies before MRI and transrectal ultrasound-guided biopsy; and (c) prebiopsy MRI in our institutions and an interval between MR examination and biopsy of less than two weeks. The exclusion criteria were as follows: (a) severe MRI artefacts and (b) incomplete MRI images or clinical information. The flow chart of the study population is shown in Fig. 1.

### MRI assessment and pathological evaluation

MRI images were interpreted by two board-certified radiologists (reader 1 and reader 2, as “expert” radiologists and “basic” radiologists, respectively, according to the criteria provided in the European Society of Urogenital Radiology (ESUR) and EAU Section of Urologic Imaging (ESUI) consensus statement [24].). The lesions were scored according to the assessment criteria provided in PI-RADS v2.1 [9]. A consensus was reached by discussion in case of disagreement. For cases with more than one suspicious lesion, the one with the highest PI-RADS assessment category was recorded. The volume of the prostate was calculated using ellipsoid formulation ([maximum anteroposterior diameter] × [maximum transverse diameter] × [maximum longitudinal diameter] × 0.52), and the diameters were measured as suggested in PI-RADS v2.1 [9]; that is, the maximum longitudinal diameter and maximum anteroposterior diameter were measured on midsagittal T2-weighted MRI, and the maximum transverse diameter was measured on axial T2-weighted MRI.

The biopsy cores were acquired by transrectal ultrasound-guided systematic biopsies (10 to 12 cores) and, in some cases, with additional MR-targeted biopsy obtained through cognitive guidance (2 to 5 cores). The biopsy cores or prostatectomy specimens were evaluated by board-certified pathologists, and the pathological diagnosis of prostate adenocarcinoma was made according to the World Health Organization (WHO) classification of tumours of the urinary system and male genital organs [25].

### MRI images acquisition and regions of interest (ROI) images acquisition

All patients underwent prostate MRI on 3.0 Tesla MR scanners (Ingenia, Phillips/ TrioTim, Siemens) without an endorectal coil. The main parameters of axial T2 weighted imaging (T2WI) for the training and internal validation set were as follows: the echo time (TE) was 80 ms, the repetition time (TR) was 4000 ms, the spacing between slices was 0 mm, the slice thickness was 3 mm, the field of view (FOV) was 180 mm × 180 mm and the voxel size was 1 × 1.1 × 3. The detailed scanning parameters are shown in Supplemental Table S1 and Table S2.

The ROI refers to the whole prostate, and was delineated by three board-certified radiologists on axial T2-weighted images. To meet the requirements of the TL model for training data, we preprocess the data of the input model to 224 × 224 × 3 images. The detailed process of data preprocessing is shown in Supplement A1.

### Building the clinical model (CM)

The CM incorporating the clinical factors and the PI-RADS score was built through the following three steps. First, we analysed inter-reader agreements (reader 1 vs. reader 2) of the PI-RADS score using Cohen’s kappa test. Second, statistical tests of the clinical parameters and the PI-RADS score were conducted using the Mann‒Whitney U test or Pearson Chi-square test. The clinical parameters included age, PSA, and prostate volume. Third, factors with statistically significant differences were selected to develop a clinical model by multivariate logistic regression with a stepwise forward selection of variables, according to Akaike’s information criterion values.

### Transfer learning feature extraction based on the transfer learning model

**Fig. 2:**
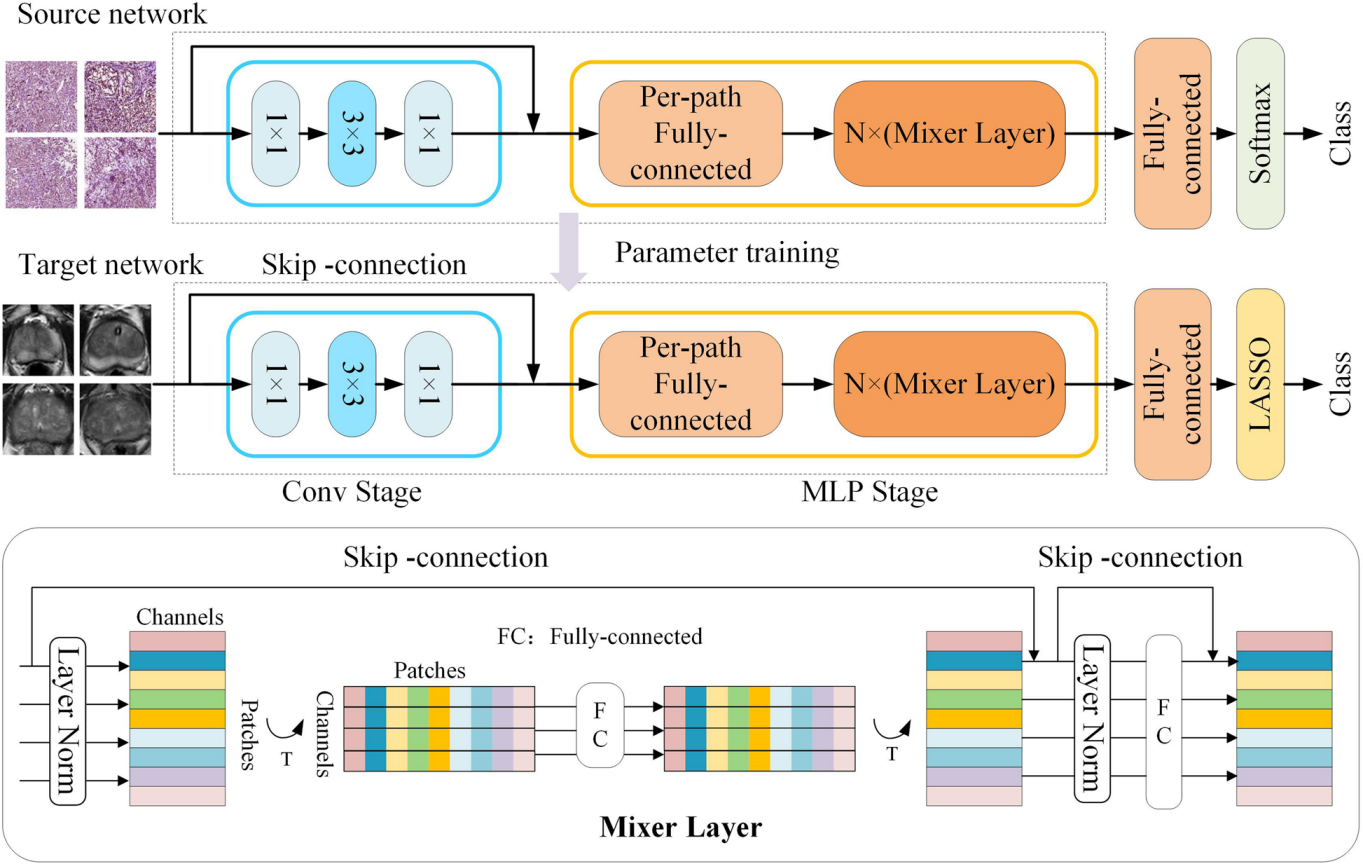
Transfer learning model based on MLP Mixer

A transfer learning model was proposed to extract the robust features of the prostate from MR images. As shown in Fig. 2, the structure of the transfer learning model was composed of a convolution block and multilayer perceptron (MLP) mixer [26]. The convolution layer is used to obtain the local features of the image. The MLP mixer layer is used for information fusion in the spatial domain and channel domain. In this study, all parameters of the pretrained model were trained by the whole slide images (WSIs) of PCa from the Cancer Genome Atlas [27], which was the source of initial weights for all subsequent models. All layers of the models were unfrozen, and the target model was trained by the dataset of MR data. The detailed training parameters of the transfer learning model are described in Supplement A2.

Based on the transfer learning model, 28,320 transfer learning features were extracted from each patient. The architecture of the transfer learning model and the process of feature extraction are shown in Supplement A3.

### Building a transfer learning signature (TLS) based on transfer learning feature

The following three steps were performed to build the TLS based on the transfer learning feature. First, the significance of each TL feature in differentiating the BCs and PCa groups was determined using the Mann‒Whitney U test. Second, the least absolute shrinkage and selection operator (LASSO) algorithm based on 10-fold cross-validation was used to select significant TL features and develop TLS. In addition, we also use support vector machine (SVM) and extreme learning machine (ELM) to develop TLS at the same time to verify the performance of the model on internal and external validation datasets. Finally, we computed the TLS score by combining the selected TL features linearly and weighting them based on their coefficients.

### Development of a transfer learning nomogram (TLN)

Multifactor logistic regression analysis based on the forward stepwise selection method [28] was used to select risk factors from TLS, age, PSA, volume and PI-RADS and construct the TLN as the final prediction model.

### Model validation index

To evaluate the performance of each model, the receiver operating characteristic (ROC) curve, area under the curve (AUC), sensitivity, specificity, accuracy, positive probability value (PPV), negative probability value (NPV), DeLong test, integrated discrimination improvement (IDI) and decision curve analysis (DCA) were calculated.

### Statistical analysis

According to reference [29], statistical analysis of the clinical factors and clinical MRI interpretation were performed.

## Results

### The CM construction

The interrater agreement of prostate volume was excellent, and the ICC value was 0.970. The Interrater agreement of PI-RADS score was medium, and the kappa coefficient was 0.578. The PI-RADS score and prostate volume were unanimously approved by the two readers. Data on clinical factors and PI-RADS scores of all cohorts are presented in Table 1. In the training cohort, the BCs and PCa groups differed significantly in factors including age, prostate volume, PI-RADS score, and PSA (*p* < 0.01, < 0.001, < 0.001, and < 0.001, respectively).

**Table 1:** Clinical factors and MRI findings of the patients

Factors	Training cohort (*n* = 309)			Internal validation cohort (*n* = 200)			External validation cohort (*n* = 200)		
	BCs	PCa	*P*-Value	BCs	PCa	*P*-Value	BCs	PCa	*P*-Value
	*n* = (184)	*n* = (125)		*n* = (112)	*n* = (88)		*n* = (79)	*n* = (121)	
**Age, years (mean ± SD)**	68.36 ± 8.27	71.06 ± 7.93	0.003	67.88 ± 7.86	71.90 ± 7.99	0.001	71.41 ± 8.81	72.37 ± 7.61	0.410
**PSA**									
100 < PSA	1	54	< 0.001	0	35	< 0.001	2	53	< 0.001
20 < PSA ≤ 100	80	45		54	31		11	47	
10 < PSA ≤ 20	70	16		37	13		24	13	
4 ≤ PSA ≤ 10	28	7		18	8		39	5	
PSA < 4	5	3		3	1		3	3	
**PI-RADS V2.1 score**									
1–2	112	8	< 0.001	64	5	< 0.001	25	5	< 0.001
3–5	72	117		48	83		54	116	
**Prostate volume**, **ml (mean ± SD)**	98.34 ± 55.01	75.39 ± 55.68	< 0.001	89.77 ± 58.90	75.97 ± 38.80	< 0.001	101.06 ± 54.95	65.90 ± 35.83	< 0.001

*BCs* benign conditions, *PCa *Prostate Cancer, *PI-RADS V2.1 *Prostate Imaging-Reporting and Data System version 2.1, *PSA *Prostate-specific antigen, *SD *Standard deviation

The clinical model was built using multivariable logistic regression, where age (odds ratio [OR], 1.051; 95% confidence interval [CI], 1.012–1.091; *p* value = 0.01), prostate volume (OR, 0.988; 95% CI, 0.980–0.995; *p* value = 0.001), PSA (OR, 2.595; 95% CI, 1.825–3.692; *p* value < 0.001) and PI-RADS score (OR,13.699; 95% CI, 6.031–31.118; *p* value < 0.001) were identified as independent risk factors (Table 2).

**Table 2:** Independent risk factors associated with prostate cancer in the clinical model by multivariate logistic regression

Intercept and variable	β	OR (95%CI)	*P* -value
Intercept	-8.188		< 0.001
Age	0.049	1.051 (1.012–1.091)	0.01
PSA	0.954	2.595 (1.825–3.692)	< 0.001
PI-RADS score	2.617	13.699 (6.031–31.118)	< 0.001
Volume	-0.012	0.988 (0.980–0.995)	0.001

*PSA* Prostate-specific antigen, *PI-RADS *Prostate Imaging-Reporting and Data System, *CI *Confidence interval, *OR *Odds ratio

### Transfer learning feature selection and TLS construction

**Fig. 3:**
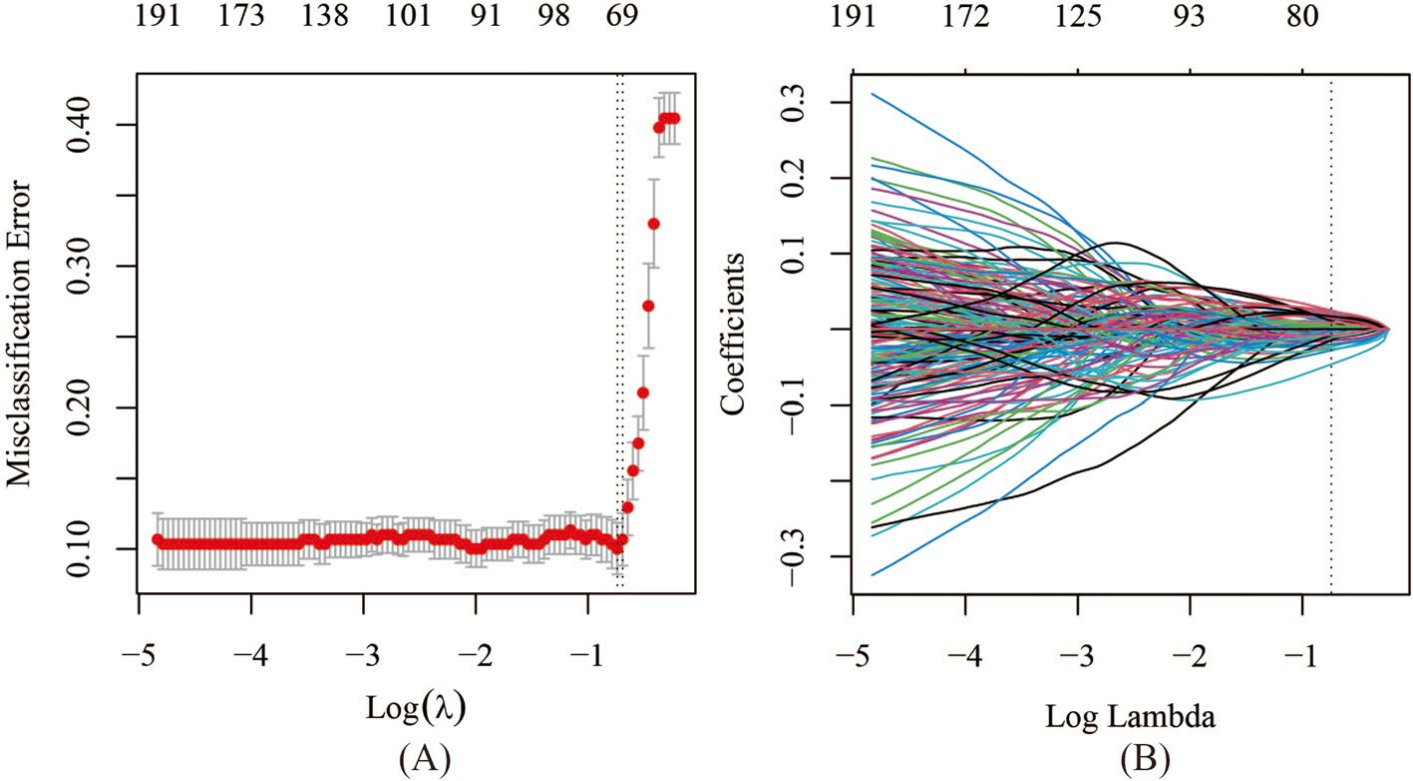
Selection by LASSO logistic regression. **a** The selection of tuning parameter (λ) using a 10-fold cross-validation according to the minimum criteria. At the optimal value of λ, the dotted vertical line was plotted. The optimal value of λ was 0.477, and log(λ)= -0.740. **b** Coefficient profiles determined by lasso logistic regression analysis of features. At log(λ)= -0.740, the dotted vertical line was drawn, including 73 optimal features with non-zero coefficients. LASSO: least absolute shrinkage and selection operator

To verify which classifier could achieve better performance, we built three TLSs with LASSO, SVM, and ELM classifiers. The value of each TL feature in differentiating the BCs and PCa groups was determined using the Mann‒Whitney U test. In total, 21,900 TL features significantly differed between the BCs and PCa groups in the training cohort. Among these, 73 TL features with nonzero coefficients were selected for inclusion in the TL score calculation formula using LASSO logistic regression (Fig. 3, Supplement A4). The detailed procedure of constructing the TLS with SVM and ELM is shown in Supplement A4.

### TLS assessment and comparison

**Fig. 4:**
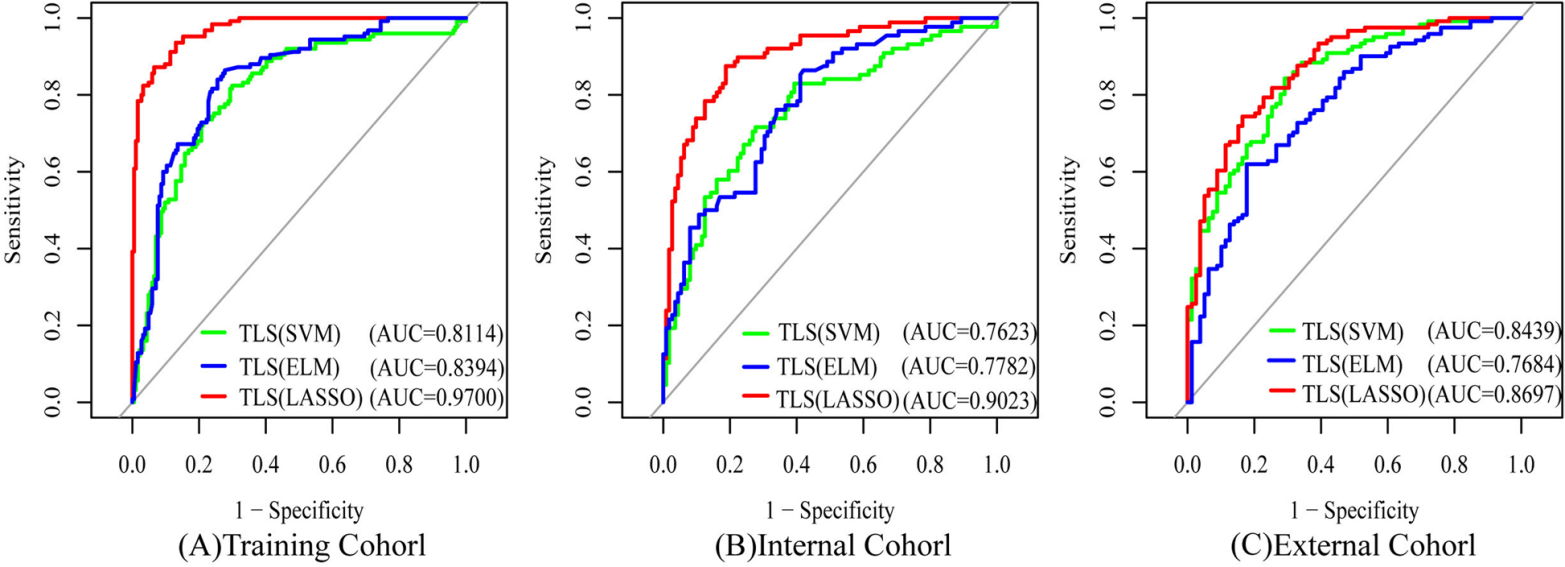
Receiver operating characteristics curves of each set using different classifier. **a** training cohort, **b** internal validation cohort, **c** external validation cohort. LASSO: transfer learning radiomics nomogram; SVM: Support Vector Machine. ELM: Extreme Learning Machine; TLN: transfer learning nomogram; TLS: transfer learning signature

Comparison of the performance of those models revealed that LASSO displayed significantly higher efficiency of diagnosis in the training (AUC = 0.9700 (95% CI, 0.9549–0.9852)), internal validation (AUC = 0.9023 (95% CI, 0.8596–0.9450)), and external validation cohorts (AUC = 0.8697 (95% CI, 0.0.8202–0.9191)) (Table 3; Fig. 4).

**Table 3:** Performance of the TLS based on LASSO, SVM, ELM classifiers in the training, internal validation, and external validation cohorts

Dataset	Models	AUC (95% CI)	Sensitivity	Specificity	Accuracy	PPV	NPV
Training cohort (*n *= 309)	TLS (LASSO)	0.9700 (0.9549–0.9852)	0.8720 (109/125)	0.9348 (172/184)	0.9094 (281/309)	0.9008 (109/121)	0.9149 (172/188)
	TLS (SVM)	0.8114 (0.7610–0.8618)	0.8240 (103/125)	0.7011 (129/184)	0.7508 (232/309)	0.6519 (103/158)	0.8543 (129/151)
	TLS (ELM)	0.8394 (0.7942–0.8846)	0.8640 (108/125)	0.7228 (133/184)	0.7799 (241/309)	0.6792 (108/159)	0.8867 (133/150)
Internal validation cohort (*n* = 200)	TLS (LASSO)	0.9023 (0.8596–0.9450)	0.7841 (69/88)	0.8750 (98/112)	0.8350 (167/200)	0.8313 (69/83)	0.8376 (98/117)
	TLS (SVM)	0.7623 (0.6944–0.8302)	0.7159 (63/88)	0.7054 (79/112)	0.7100 (142/200)	0.6563 (63/96)	0.7596 (79/104)
	TLS (ELM)	0.7782 (0.7149–0.8146)	0.6932 (61/88)	0.6875 (77/112)	0.6900 (138/200)	0.6354 (61/96)	0.7404 (77/104)
External validation cohort (*n *= 200)	TLS (LASSO)	0.8697 (0.8202–0.9191)	0.6446 (78/121)	0.8861 (70/79)	0.7400 (148/200)	0.8966 (78/87)	0.6195 (70/113)
	TLS (SVM)	0.8439 (0.7899–0.8980)	0.8182 (99/121)	0.7215 (57/79)	0.7800 (156/200)	0.8182 (99/121)	0.7215 (57/79)
	TLS (ELM)	0.7684 (0.7014–0.8354)	0.6198 (75/121)	0.8228 (65/79)	0.700 (140/200)	0.8427 (75/89)	0.5856 (65/111)

*LASSO* Least absolute shrinkage and selection operator, *SVM *Support Vector Machine, *ELM *Extreme Learning Machine, *AUC *Area under the curve, *CI *Confidence interval, *TLS *Transfer learning signature, *PPV* Positive predictive value, *NPV *Negative predictive value

### TLN construction and validation

**Fig. 5:**
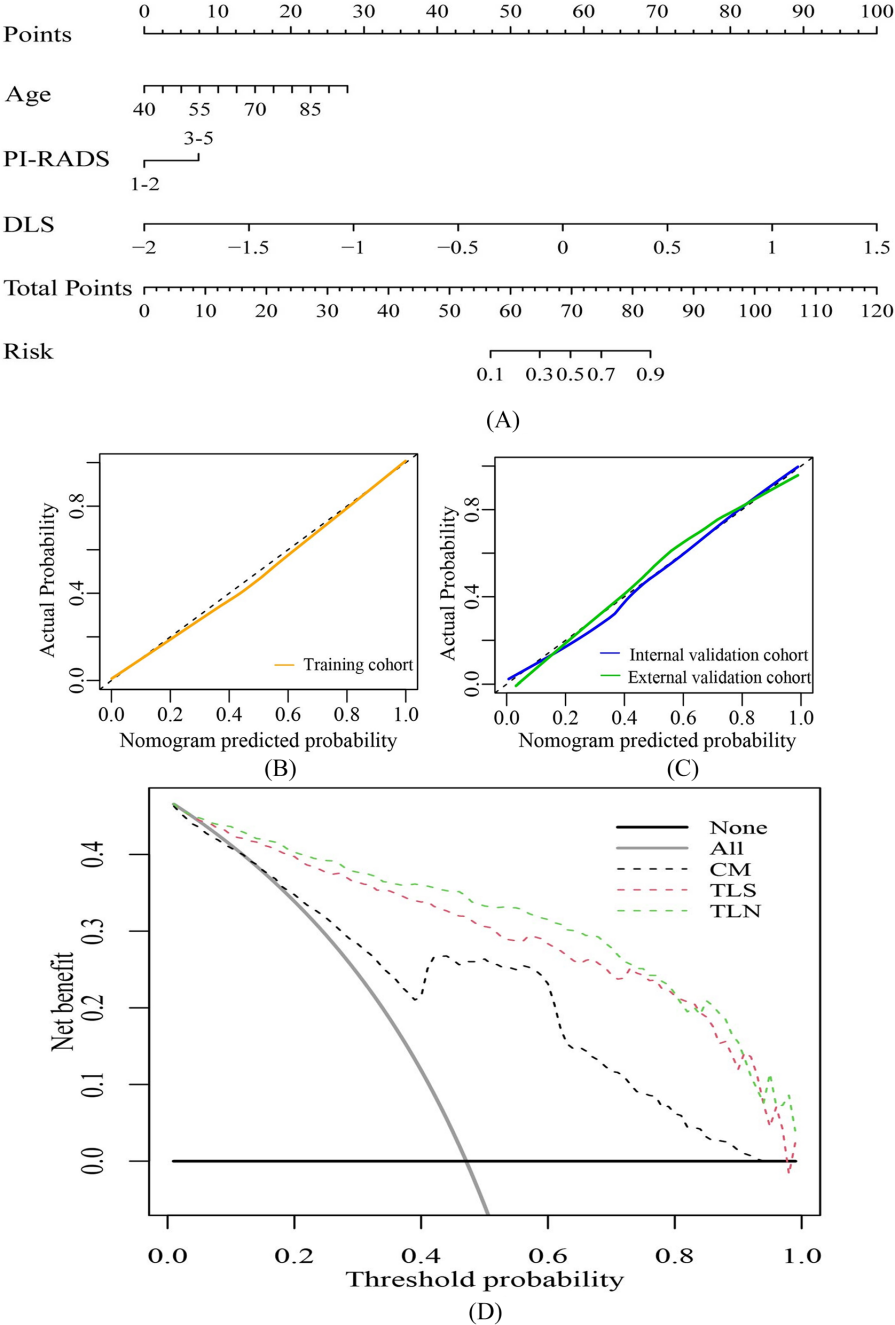
Construction of the TLN and DCA of the various diagnostic models. **a** The presented TLN that incorporates both TL signature and clinical factors. Calibration curves of the TLS in training (**b**) and two validation cohorts (**c**), respectively. **d **DCA of different diagnostic models. The solid gray and black lines indicate the assumption that all and none of the PCa groups are involved, respectively. The threshold probability was defined as the point at which the expected benefit of the treatment was equal to the benefit of avoiding treatment. The results indicated that the TLN provided a greater net benefit than the clinical model and TLS (range 0.01– 0.99). TLN: transfer learning nomogram; TLS: transfer learning signature; DCA: decision curve analysis

**Fig. 6:**
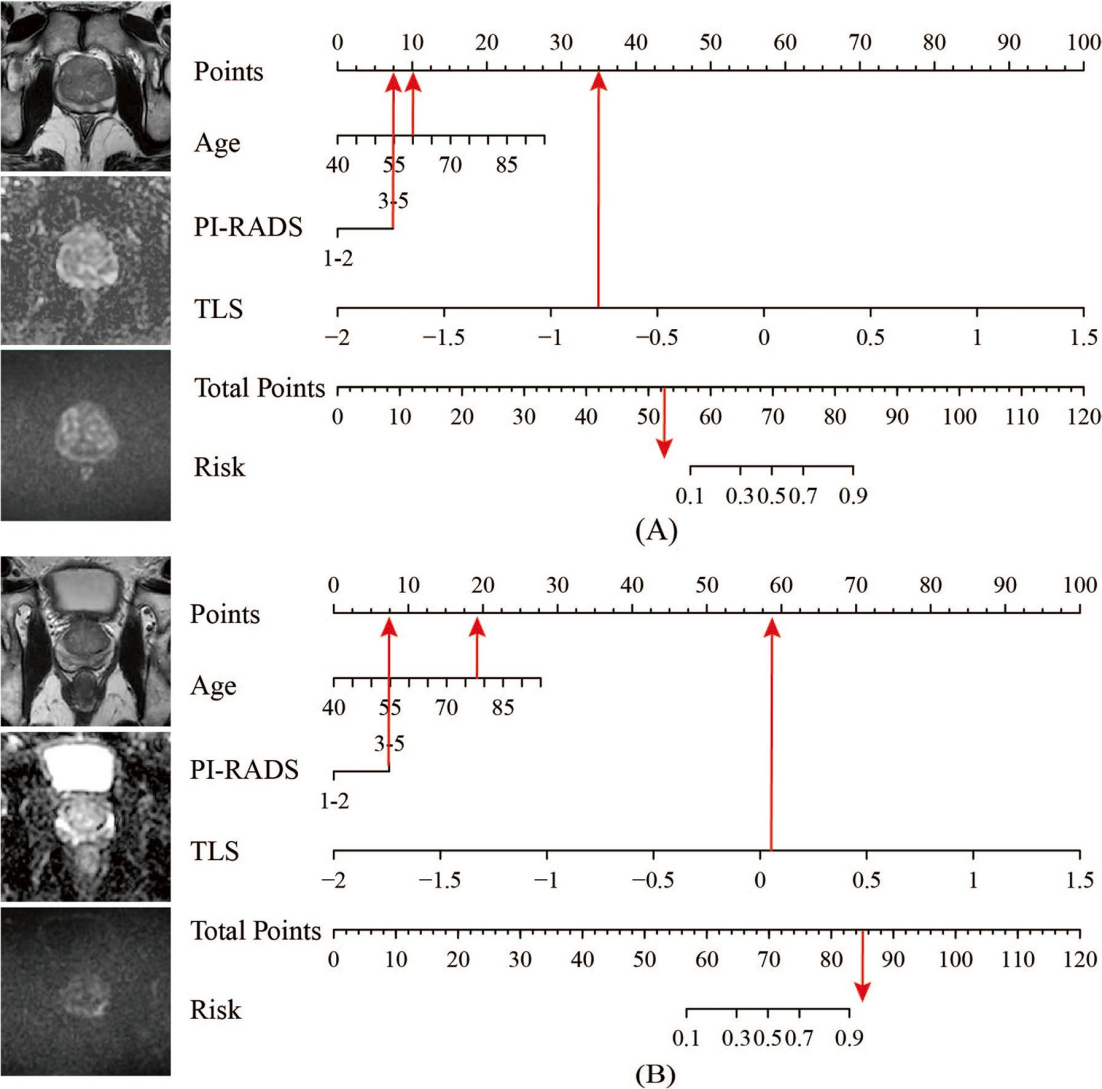
Examples of the nomogram in clinical practice. **a** A patient with raised prostate-specific antigen and Prostate Imaging Reporting and Data System category 3 lesion in left medial posterior peripheral zone. The total score is 52.5, which corresponds to a prostate cancer risk of less than 0.1. The biopsy result was benign. **b** A patient with raised prostate-specific antigen and Prostate Imaging Reporting and Data System category 3 lesion in left medial posterior peripheral zone. The total score is 85.1, which corresponds to a prostate cancer risk of greater than 0.9. The biopsy result was Gleason grade group 2 prostate cancer. TLS: transfer learning signature

In the multivariable logistic regression analysis, TLS (OR, 120.780; 95% CI, 34.648–421.028; *p* value < 0.001), age (OR, 1.088; 95% CI, 1.030–1.150; *p* value = 0.003), and PI-RADS score (OR, 3.472; 95% CI, 1.198–10.058; *p* value = 0.022) were independent predictors (Table 4). Incorporating these three independent factors, we constructed a combined model to be presented as a transfer learning nomogram (Fig. 5a). Examples of the clinical use of the nomogram are shown in Fig. 6.

**Table 4:** The parameters of the TLN for BCs and PCa in patients of the training set

Intercept and variable	β	OR(95%CI)	*P* -value
Intercept	-5.513		0.005
Age	0.085	1.088 (1.030–1.154)	0.003
PI-RADS score	1.245	3.472 (1.198–10.058)	0.022
TLS	4.794	120.780 (36.648-421.028)	< 0.001

*PI-RADS* Prostate Imaging-Reporting and Data System, *TLS *Transfer Learning Signature, *CI *Confidence interval, *OR *Odds ratio

Using the calibration curve, we confirmed a marked connection between the predicted and actual data in the training cohort (Fig. 5b and c).

**Fig. 7:**
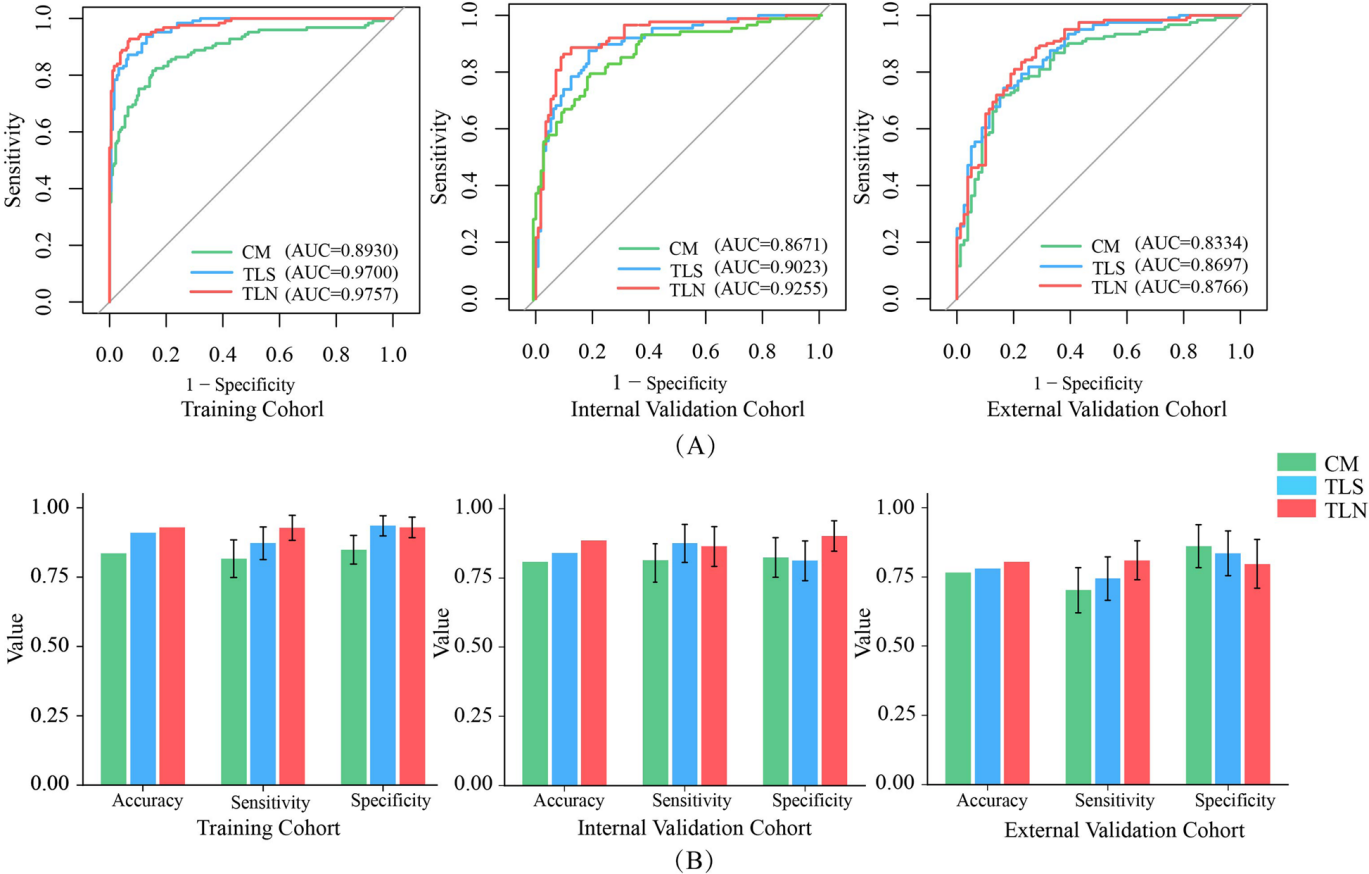
Comparison of the performance of the CM, TLS and TLN. **a** Receiver operating characteristics curves of three models in each set; **b** Diagnostic performance of the three models. TLN: transfer learning nomogram; TLS: transfer learning signature. CM: clinical model

The diagnostic performance of the CM, TLS, and TLN are shown in Table 5; the ROC curves of these three models are shown in Fig. 7. In the internal and external validation cohorts, respectively, the transfer learning nomogram achieved the best discrimination (AUC, 0.9255; 95% CI, 0.8873–0.9638 and AUC, 0.8766; 95% CI, 0.8267–0.9264, accuracy of 0.8750 and 0.7700, sensitivity of 0.9280 and 0.7273, and specificity of 0.9293 and 0.8354).

**Table 5:** Performance of the clinical model, TLS, and TLN in the training, internal validation, and external validation cohorts

Dataset	Models	AUC (95% CI)	Sensitivity	Specificity	Accuracy	PPV	NPV
Training cohort (*n* = 309)	CM	0.8930 (0.8539–0.9320)	0.8160 (102/125)	0.8478 (156/184)	0.8350 (258/309)	0.7846 (102/130)	0.8715 (156/179)
	TLS	0.9700 (0.9549–0.9852)	0.8720 (109/125)	0.9348 (172/184)	0.9094 (281/309)	0.9008 (109/121)	0.9149 (172/188)
	TLN	0.9757 (0.9613–0.9902)	0.9280 (116/125)	0.9293 (171/184)	0.9288 (287/309)	0.8992 (116/129)	0.9500 (171/180)
Internal validation cohort (*n* = 200)	CM	0.8671 (0.8158–0.9184)	0.7955 (70/88)	0.8036 (90/112)	0.8000 (160/200)	0.7609 (70/92)	0.8333 (90/108)
	TLS	0.9023 (0.8596–0.9450)	0.7841 (69/88)	0.8750 (98/112)	0.8350 (167/200)	0.8313 (69/83)	0.8376 (98/117)
	TLN	0.9255 (0.8873–0.9638)	0.8636 (76/88)	0.8839 (99/112)	0.8750 (175/200)	0.8539 (76/89)	0.8919 (99/111)
External validation cohort (*n* = 200)	CM	0.8334 (0.7752–0.8915)	0.8017 (97/121)	0.7089 (56/79)	0.7650 (153/200)	0.8083 (97/120)	0.7000 (56/80)
	TLS	0.8697 (0.8202–0.9191)	0.6446 (78/121)	0.8861 (70/79)	0.7400 (148/200)	0.8966 (78/87)	0.6195 (70/113)
	TLN	0.8766 (0.8267–0.9264)	0.7273 (88/121)	0.8354 (66/79)	0.7700 (154/200)	0.8713 (88/101)	0.6667 (66/99)

*AUC* Area under the curve, *CI *Confidence interval, *CM *Clinical model, *TLS *Transfer learning signature, *TLN *Transfer learning nomogram, *PPV *Positive predictive value, *NPV *Negative predictive value

The confusion matrix of the TLN is presented in Table 6.

**Table 6:** Confusion matrix for predicted versus actual categories

		Predicted result	
		BCs	PCa
Gold standard	BCs	336	39
	PCa	54	280

*BCs* Benign conditions, *PCa* Prostate Cancer

The Delong test and IDI demonstrated that the TLN had significantly superior performance to both the TLS and CM in all validation cohorts (Delong test, *P* < 0.001, *P* = 0.0263; IDI = 0.0611, *P* < 0.001, IDI = 0.1272, *P* < 0.001, respectively).

The decision curve showed that the TLN added more net benefit than the CM in differentiating the BCs and PCa groups within the range of threshold probability 0.01 to 0.99 (Fig. 5d).

## Discussion

In the present study, we developed and validated an integrated nomogram incorporating useful TL features, clinical MRI interpretation, and clinical predictors for PCa differentiation. By comparison, the TLN outperformed TLS alone or CM in both the internal and external validation cohorts. The proposed TLN could be a better noninvasive diagnostic tool for differentiating PCa and BCs.

PI-RADS score was identified as an independent risk factor in both CM and TLN. In clinical MRI interpretation, PI-RADS score assignment is evaluated by radiologists based on MRI findings. T2-weighted images are often used to display anatomical information, detect abnormalities and evaluate extraprostatic extension. The apparent diffusion coefficient (ADC) map and high b-value images of diffusion-weighted imaging (DWI) are valuable for lesion detection since prostate cancers often have restricted water molecule diffusion. DCE can be used to detect some small cancers with early enhancement. For the peripheral zone, DWI is the primary determining sequence, and the T1-weighted dynamic contrast material–enhanced (DCE) result can be helpful when the DWI score is intermediate. For the transitional zone, the T2-weighted score is the dominant factor, and the DWI score is also useful in atypical transitional zone nodules. As a standardized risk assessment tool for PCa and csPCa, the PI-RADS score has been incorporated by some prediction models [30, 31] and performed better than those without mp-MRI.

In addition to PI-RADS score assignment, PSA, prostate volume, and age were identified as independent risk factors in our CM and were also significant predictors of PCa or csPCa in previous studies [29, 30]. However, these predictors are not cancer specific; benign conditions, such as benign prostatic hyperplasia, may also result in the enlargement of the prostate and an increase in PSA and are more prevalent in ageing men [32], which may explain why CM performed less strongly in both internal and external validation cohorts than TLS and TLN when differentiating PCa and BCs.

During the TLS construction, we applied TL to mitigate the overfitting in the process of model training using small data and extracting robust features. We chose the WSIs of PCa as the source domain dataset for TL because they are medical images containing tumour histopathological information and are more similar to prostate magnetic resonance images than natural images. Literatures have reported the correlation between prostate MRI characteristics and histological conditions presented in histopathological slides [12, 33]. The more similar the source domain data are to the target domain data, the more a small training dataset can make full use of the transfer of learning [34, 35]. Our TL method can autonomously mine features based on the WSIs of PCa from superficial to deep layers of the image through multilayered networks. These features were more relevant to the task, contained more lesion information, and were essential for differentiating PCa and BCs. The proposed TLS also performed well in the internal and external validation cohorts.

The final prediction model TLN was constructed by combining TLS, age, and PI-RADS score. The performance of the TLN was encouraging, with AUCs of 0.9255 and 0.8766 in the two validation cohorts. Furthermore, we found that the performance of TLN was better than that of CM or TLS alone. This may be because not only imaging features but also clinical factors as well as clinical MRI interpretation were taken into account when constructing the TLN.

The current study has some limitations. First, the sample size used in this research was still limited, and further multicentre validation is needed before the TLN can be used routinely in clinical practice. Second, for most patients, the pathological assessment was based on biopsy cores acquired by transrectal ultrasound-guided biopsies, which may lead to the missed diagnosis of some undetected PCa. Third, our model was restricted to differentiating PCa from BCs, and did not encompass the differentiation between csPCa and clinically insignificant prostate cancer. We are considering developing a reliable model to predict csPCa in future work.

## Conclusions

In conclusion, we developed a TLN that could be an important risk assessment tool for the differentiation of PCa and BCs, providing valuable assistance in clinical decision-making.

### Supplementary Information

**Additional file 1.**

## Data Availability

The datasets used and/or analysed during the current study are available from the corresponding author on reasonable request.

## Abbreviations

ADC: Apparent diffusion coefficient
AUC: Area under the curve
BCs: Benign conditions
CM: Clinical model
csPCa: Clinically significant prostate cancer
DCA: Decision curve analysis
DCE: Dynamic contrast material–enhanced
DL: Deep learning
DRE: Digital rectal examination
DWI: Diffusion-weighted imaging
ELM: Extreme Learning Machine
FOV: Field of view
IDI: Integrated discrimination improvement
ISUP: International Society of Urological Pathology
LASSO: Least absolute shrinkage and selection operator
ML: Machine learning
MLP: Multi-layer perceptron
mp-MRI: Multi-parametric magnetic resonance imaging
MRI: Magnetic resonance imaging
NPV: Negative probability value
PCa: Prostate cancer
PI-RADS: Prostate Imaging Reporting and Data System
PPV: Positive probability value
PSA: Prostate specific antigen
ROC: Receiver operating characteristic curve
SVM: Support Vector Machine
T2WI: T2 weighted imaging
TE: Echo time
TLN: Transfer learning nomogram
TL: Transfer learning
TLS: Transfer learning signature
TR: Repetition time
WSIs: Whole slide images
