# Supplementary material for: A transfer learning nomogram for predicting prostate cancer and benign conditions on MRI
Source: BMC Med Imaging. 2023 Nov 30;23:200. doi: 10.1186/s12880-023-01163-7 (PMC10691068; doi:10.1186/s12880-023-01163-7)
Supplement: Supplementary file 1 — Additional file 1. [file 12880_2023_1163_MOESM1_ESM.doc]

Supplementary material

Supplement A1 MR image preprocessing

On the T2 weighted imaging, the region of interest was intercepted according to the prostate coordinates given by the radiologist. The interception rule of the region of interest was to take the coordinate point as the center and expand 64 pixel screenshots to the up, down, left and right directions of the coordinate point respectively, the size of captured image was 128 × 128 square. All images were resized to 224 × 224 pixels by interlinear interpolation using OpenCV-python 3.6 to fit the format size of our model input.

Supplement A2 Training process of deep learning model

Our method was implemented in Python (version 3.6) and performed on a machine with an Intel Core i7-9700X CPU @ 3.60GHz. The model training was implemented using GPU-torch and was accelerated on an NVIDIA GeForce RTX 2080(8 GB on-board memory).

The network training was trained by stochastic gradient descent with a momentum of 0.9. We used a weight decay of 1e-4 and an initial learning rate 0.0001. For all experiments, we trained 150 epochs. The size of batch was 16 for small image experiments. The Cross-Entropy-Loss was used as the loss function.

Supplement A3 The framework of the transfer learning model and the process of feature extraction

We propose a transfer learning model combining convolution and multi-layer perceptron(MLP), which takes the preprocessed MR images as input. The model consists of two convolution blocks which are composed of 1×1, 3×1 and 1×1 convolution kernels and 12 MLP mixer layers. The shallow layer of our MLP structure introduces two convolution blocks connected by residues, uses convolution layers to extract sufficient local features, and avoids the pretraining requirement of MLP mixer on large-scale datasets, while MLP mixer layers captures information of prostate MR images on spatial domain versus channel domain, which is essential for differential diagnosis of prostate tumors.

We use the above model to extract MR images features. Firstly, input the preprocessed pictures into the trained deep learning model. Secondly, the MLP mixer layer was used as the feature extractor to average the eigenvalues obtained after the picture passes through token-mixing MLP and channel-mixing MLP. Finally, 28,230 deep learning features were extracted from each patient by stitching the eigenvalues extracted from mixer layer.

Supplement A4 Transfer learning signature score calculation

The transfer learning model was constructed by using the deep learning features of 73 non-zero coefficients determined by lasso logistic regression, and the deep learning signature score of each patient was calculated. The calculation method adopts linear weighting strategy and non-zero coefficient as the corresponding weight. The calculation formula of deep learning signature score is as follows:

In the above formula, $S$represents deep learning signature score; represents the $i$weight; represents the $i$deep learning feature. The same strategy was used to calculate the deep learning score of each phase; b represents bias.

Table S1. Prostate MRI protocol of internal cohort 1

| Sequence | Orientation | TR (ms) | TE (ms) | Slice  Thickness (mm) | Spacing  Between Slices (mm) | FOV (mm)  frequency × phase | Matrix | Additional features |
| --- | --- | --- | --- | --- | --- | --- | --- | --- |
| T2W_TSE | transverse | 4000 | 80 | 3 | 0 | 180×180 | 180×159 |  |
| T2W_TSE | sagittal | 1250 | 80 | 4 | 0.5 | 260×220 | 236×199 |  |
| T2W_TSE | coronal | 1250 | 80 | 4 | 0.6 | 261×225 | 236×205 |  |
| T2W_SPAIR | transverse | 4000 | 80 | 3 | 0 | 180×180 | 180×159 |  |
| T1W_TSE | transverse | 913 | 8 | 3 | 0 | 180×180 | 224×177 |  |
| DWI | transverse | 5292 | 100 | 3 | 0 | 180×180 | 70×70 | b-factors 0/100/800/1000/  1500/2000 |
| DYNAMIC | transverse | 4 | 2 | 3.5 | 0 | 180×180 | 128×100 |  |

TSE = turbo-spin-echo; SPAIR = spectral adiabatic inversion recovery; TR = repetition time, TE = echo time, FOV = field of view

Table S2. Prostate MRI protocol of external cohort 2

| Sequence | Orientation | TR (ms) | TE (ms) | Slice  Thickness (mm) | Dist factor% | FOV read (mm)/FoV phase % | Matrix | Additional features |
| --- | --- | --- | --- | --- | --- | --- | --- | --- |
| T2W_TSE | transverse | 3640 | 109 | 3 | 0 | 230/90.6 | 256×347 |  |
| T2W_TSE | sagittal | 3630 | 108 | 3 | 10 | 250/100 | 313×357 |  |
| T2W_TSE | coronal | 4820 | 67 | 3 | 10 | 380×85.0 | 238×269 |  |
| T2W_TSE_FS | transverse | 4870 | 87 | 3 | 0 | 230/90.6 | 256×347 |  |
| T1_TSE | transverse | 553 | 13 | 6 | 20 | 330/75.0 | 220×248 |  |
| EP2D_DIFF | transverse | 5200 | 92 | 3 | 0 | 260/85.0 | 113×138 | b-factors 0/2000 |

TSE = turbo-spin-echo; FS= fat suppression; TR = repetition time, TE = echo time, FOV = field of view
